# The Maintaining Musculoskeletal Health (MAmMOTH) Study: Protocol for a randomised trial of cognitive behavioural therapy versus usual care for the prevention of chronic widespread pain

**DOI:** 10.1186/s12891-016-1037-4

**Published:** 2016-04-26

**Authors:** Gary J. Macfarlane, Marcus Beasley, Gordon Prescott, Paul McNamee, Philip Keeley, Majid Artus, John McBeth, Philip Hannaford, Gareth T. Jones, Neil Basu, John Norrie, Karina Lovell

**Affiliations:** Epidemiology Group, School of Medicine, Medical Sciences and Nutrition, University of Aberdeen, Aberdeen, UK; Aberdeen Centre for Arthritis and Musculoskeletal Health, University of Aberdeen, Aberdeen, UK; Medical Statistics Team, Institute of Applied Health Sciences, University of Aberdeen, Aberdeen, UK; Health Economics Research Unit, Institute of Applied Health Sciences, University of Aberdeen, Aberdeen, UK; Department of Health Sciences, University of Huddersfield, Huddersfield, UK; Arthritis Research UK Primary Care Centre, Primary Care Sciences, Keele University, Keele, UK; Arthritis Research UK Epidemiology Unit, University of Manchester, Manchester, UK; Centre of Academic Primary Care, Institute of Applied Health Sciences, University of Aberdeen, Aberdeen, UK; Centre for Healthcare Randomised Trials (CHaRT), Health Services Research Unit, University of Aberdeen, Aberdeen, UK; School of Nursing, Midwifery and Social Work, University of Manchester, Manchester, UK

**Keywords:** Chronic widespread pain, Fibromyalgia, Prevention, Randomised trial, Treatment as usual, Usual care, Chronic pain, CBT, Health economics, Cost-effectiveness

## Abstract

**Background:**

Cognitive behavioural therapy (CBT) has been shown to improve outcomes for patients with fibromyalgia, and its cardinal feature chronic widespread pain (CWP). Prediction models have now been developed which identify groups who are at high-risk of developing CWP. It would be beneficial to be able to prevent the development of CWP in these people because of the high cost of symptoms and because once established they are difficult to manage. We will test the hypothesis that among patients who are identified as at high-risk, a short course of telephone-delivered CBT (tCBT) reduces the onset of CWP. We will further determine the cost-effectiveness of such a preventative intervention.

**Methods:**

The study will be a two-arm randomised trial testing a course of tCBT against usual care for prevention of CWP. Eligible participants will be identified from a screening questionnaire sent to patients registered at general practices within three Scottish health boards. Those returning questionnaires indicating they have visited their doctor for regional pain in the last 6 months, and who have two of, sleep problems, maladaptive behaviour response to illness, or high number of somatic symptoms, will be invited to participate. After giving consent, participants will be randomly allocated to either tCBT or usual care. We aim to recruit 473 participants to each treatment arm. Participants in the tCBT group will have an initial assessment with a CBT therapist by telephone, then 6 weekly sessions, and booster sessions 3 and 6 months after treatment start. Those in the usual care group will receive no additional intervention. Follow-up questionnaires measuring the same items as the screening survey questionnaire will be sent 3, 12 and 24 months after start of treatment. The main outcome will be CWP at the 12 month questionnaire.

**Discussion:**

This will be the first trial of an intervention aimed at preventing fibromyalgia or CWP. The results of the study will help to inform future treatments for the prevention of chronic pain, and aetiological models of its development.

**Trial registration:**

ClinicalTrials.gov ID: NCT02668003URL: Please check that the following URLs are working. If not, please provide alternatives: NCT02668003Alternative is: https://www.clinicaltrials.gov/ct2/show/NCT02668003>. Date registered: 28-Jan-2016.

## Background

Chronic widespread pain (CWP), the cardinal feature of fibromyalgia, is associated with lost work productivity, psychological ill health, and poor quality of life. It is one of the most common reasons for referral to a rheumatologist [1]. The cost of CWP is high in terms of both individual, societal and health costs: for example, in the United States, mean per-patient costs (including pain and non–pain-related medication, physician consultations, tests and procedures, and emergency department visits) in the 6 months following a new diagnosis of fibromyalgia have been reported as $3481, comparable to patients with rheumatoid arthritis [2] but resulting in worse quality of life [3]. Current guidelines recommend pharmacological, physical, and psychological therapies although the importance attributed to individual therapies is inconsistent [4]. There is good evidence for musculoskeletal pain conditions generally that the longer the duration of symptoms, the less likely that symptoms are to improve [5, 6], including with specific interventions [7, 8]. This is particularly so for CWP which, once developed, is challenging to manage and effect improvement.

**Fig. 1:**
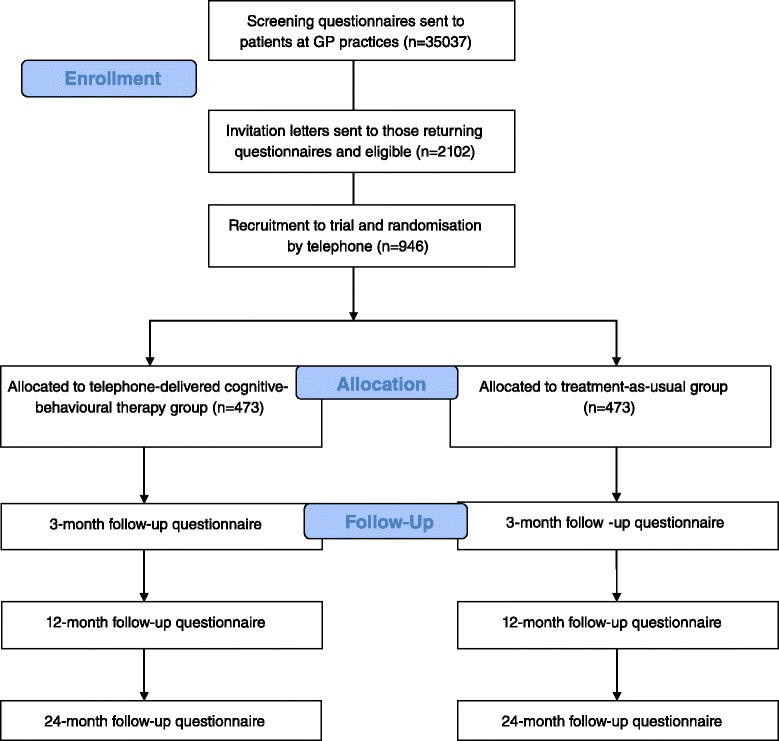
Flowchart of the study

A systematic review and meta-analysis of randomised controlled trials (RCT) of Cognitive Behaviour Therapy (CBT) for patients with fibromyalgia concluded that CBT improves coping with pain, reduces depressed mood and healthcare-seeking behaviour in such patients [9]. The delivery of CBT by telephone has been shown to be effective, acceptable and accessible [10]. The MUSICIAN study, which we have recently concluded, tested telephone-delivered CBT (tCBT) and/or exercise for patients with chronic widespread pain consulting to their GP (using a 2 × 2 factorial design). Three months after the end of therapy, both interventions resulted in significantly better primary outcome measures (patient global health) than treatment as usual (tCBT 33 % of participants with positive outcome, exercise 24 %, treatment as usual 8 %), but there was no significant additional benefit of receiving both interventions (combined 37 %). Recent analyses have demonstrated these benefits are maintained 2 years after the end of therapy (tCBT 35 % with positive outcome, exercise 29 %, combined 31 %, treatment as usual 13 %), and that tCBT is highly cost-effective [11].

We have conducted a comprehensive literature review with the aim of identifying randomised trials which had the aim of preventing the onset either of CWP or fibromyalgia. This review did not identify any such published trials. Further, a search of 11 international clinical trials registers/databases (including US, UK, Europe, Australia/New Zealand, Japan), undertaken in Autumn 2013 did not identify any ongoing trial with the aim of preventing the onset of CWP (or fibromyalgia). There are several reasons why it may be desirable to try to prevent CWP onset, namely that the majority of CWP patients do not have important symptom improvement with current management (even within trials). Prediction models from epidemiological studies have been developed to identify “high-risk” patients, which makes such an approach feasible. Research using the General Practice Research Database has demonstrated that prior to receiving a diagnosis of fibromyalgia in primary care, persons have a long-term prior history of consultation with symptoms [12]. Although this will be the first prevention trial in this area, the concept of prevention using CBT has been addressed in musculoskeletal disorders with respect to intervention in neck pain and low back pain before people become patients [13] and in mental disorders [14].

We have conducted prospective epidemiological studies which have demonstrated that it is possible to identify “high-risk” groups. In the first study, a high risk group for CWP onset was identified on the basis of two factors: somatic awareness (using the Somatic Symptom Scale) and illness behaviour (using the Illness Behaviour Score) [15]. This was replicated in a second study conducted by the applicants [16]. These “aetiological models” excluded pain and therefore we have re-analysed data from the latter study (also considering pain status) to identify the best predictors of onset and which results in a model more suitable for use in prevention studies. The resulting “at risk model” requires regional pain and two of the following: maladaptive behavioural response to illness (Illness Behaviour score > 4), a high number of somatic symptoms (Somatic Symptom Score > 2) and sleep disturbance (Sleep Problem Scale Score > 4). In the second “validation” study, from a population of 2,374 persons without CWP, 653 satisfied the definition of “high-risk of CWP” of whom 139 had developed CWP twelve months later (that is a Positive Predictive value of 21.3 %). Amongst persons not deemed to be at high risk (*n* = 1721), 77 developed CWP which is a Negative Predictive Value of 95.5 %.

An Arthritis Research UK report on fibromyalgia/CWP, based on a think-tank held in July 2012, identified prevention as a research priority. We have previously shown short and long-term effectiveness of tCBT for CWP (compared to usual care), in an Arthritis Research UK funded study [11, 17]. Specifically this demonstrated sustained improvement in patient global assessment of change, reduced psychological distress, fear of movement and reliance on passive coping styles. Secondly we have developed and refined statistical models which identify persons at high risk for the future development of CWP. We therefore now propose a study to test whether tCBT can reduce the risk of CWP onset amongst those at high risk.

We will test the hypothesis that among patients who report regional pain for which they have already sought a consultation in primary care, and who are identified as high risk of developing chronic widespread pain, a short course of telephone-delivered Cognitive Behaviour Therapy (tCBT) reduces the onset of CWP. We will further determine the cost-effectiveness of such a preventative intervention.

## Methods

### Study design

The study will be a two-arm randomised controlled trial testing a course of tCBT against usual care among patients identified at high-risk developing CWP (Fig. 1).

### Recruitment

We will mail a randomly selected sample of adults aged 25 years and over registered with participating general practices in the study areas (NHS Grampian, NHS Highland, and NHS Greater Glasgow and Clyde). We will require the involvement of 7 or 8 general practices. The Scottish Primary Care Research Network (SPCRN) will be involved in recruitment of patients to this study from primary care. SPCRN staff will undertake searches of GPs electronic databases to identify a random sample of potentially eligible patients. Screening survey questionnaires will then be sent out by Health Informatics Centre Services in Dundee on behalf of the practice. Patients will return completed survey questionnaires to the research team at the University of Aberdeen where they will be assessed for eligibility and sent letters inviting them to the trial if eligible.

The “screening questionnaire” will determine whether the patient a) meets the study eligibility criteria and b) would be willing to be contacted again regarding a treatment trial for “musculoskeletal health”. The questionnaire will include:

- Pain assessed by specific questions on the experience of pain, consultation and body manikins (which will provide site and also allow us to exclude those who already have chronic widespread pain)
- Illness Behaviour Scale [18]
- Somatic Symptoms Scale (excluding pain items) [19]
- Sleep Problem Scale [20]
- Quality of Life and Wellbeing (EQ-5D-5L [21]; ICECAP [22])
- General Health Questionnaire (23)
- Chalder Fatigue Scale (24)

The inclusion criteria are:

- A ‘high-risk’ profile for developing CWP as identified on the screening survey, i.e.:
  - o Have pain for which they have sought consultation to primary care in the last 6 months
  - o Any 2 of the following:
    - ■ Illness Behaviour Scale Score > 4
    - ■ Somatic Symptom Scale Score > 2
    - ■ Sleep Problem Scale Score > 4
- Access to a landline or mobile telephone
- Ability to understand English sufficiently to participate in the intervention
- Ability to give informed consent
- Aged 25 years or over

Exclusion criteria:

- Meeting American College of Rheumatology definition of CWP in the 1990 criteria for fibromyalgia (as assessed by the screening questionnaire) [23]
- Medical conditions which would make the proposed intervention unsuitable (e.g. cognitive ability)
- Non-availability for the intervention

A list of patients will be available to the general practitioner in advance, with the option of indicating any as unsuitable for the study. Patients would then be sent information about the study and subsequently contacted by a member of the research team by telephone and, if appropriate, consented and recruited into the trial.

### Consent

Included in the mailed invitation to eligible patients will be an information sheet, consent form to take part in the trial, and a best-time-to-call slip. Once a patient has returned a signed consent form and best-time-to-call slip, a member of the research team will phone them. The researcher will read out from a script giving information about the study, and the patient will have the opportunity then to ask questions about the study. If the patient consents to participate they will be recruited to the study and randomised to one of the arms of the trial. (It is only at this point that the participant is considered to have given informed consent).

### Randomisation

After consent has been given to participate in the study, during the call with the participant, the researcher will contact the Trial randomisation centre at the Centre for Healthcare Randomised Trials at the University of Aberdeen, using the internet or by telephone. This service is available 24 h a day. Using a computer randomisation program, subjects will be randomly allocated into one of the two treatment groups, stratified in blocks by one predictor of outcome i.e. the number of non-pain “high-risk” factors they report (2 or 3) since this is related to the risk of CWP onset, and GP Practice. Subjects will be notified of the outcome of allocation (i.e. to which treatment group they have been assigned) during the consent/randomisation phone-call. If allocated to the active intervention, the participant will receive a phone call from a therapist to arrange an initial appointment. We will confirm the allocation to treatment arm in a letter to the participant.

### Follow up

Follow-up questionnaires will be mailed to participants at 3, 12 and 24 months after the treatment start date (for participants in the active treatment group) or dummy treatment start date (for those in usual care). The dummy treatment start date will be based on the treatment start date of the last participant to be assigned to active treatment. Instruments included in the follow-up questionnaires will be the same as in the screening survey questionnaire. Additionally, follow-up questionnaires will include the Patient Global Impression of Change (7-item scale from “very much worse” to “very much better”), and questions on health care usage.

### Treatment protocol

The CBT intervention, delivered by telephone, will consist of an initial assessment (45–60 min), 6 weekly sessions (each 30–45 min) over six weeks, and then booster sessions at 3 and 6 months. The intervention will be delivered by therapists trained for the study and accredited by the British Association for Behaviour and Cognitive Psychotherapies. Participants will be supported by a self-management CBT manual refined from the manual we developed for the MUSICIAN study where it was successfully used with demonstrable patient benefit.

There will be a patient-centred assessment by the therapist for problem identification, risk assessment and development of a shared formulation of the current health problem. The sessions will involve education about musculoskeletal pain (all persons will have recently consulted to primary care with regional pain), somatic symptoms and specific CBT techniques such as pacing of activity, behavioural activation, diary keeping, identifying and challenging negative and unhelpful thinking patterns and the development of a longer term management plan. Participants’ self-management manuals will include agreed collaborative goals for the therapist and patient to work towards, diaries, and some exercises to complete after specific sessions.

Therapists delivering the intervention will receive a 2 day training programme conducted by the investigators. Therapists will be supervised two weekly throughout the trial period by members of the trial team. Patient adherence will be examined through collecting data on number of telephone consultations conducted and evaluation of the use of CBT techniques through exercises contained in the manual.

The group allocated to usual care will receive no additional intervention – this will reflect the fact there is no specific intervention provided to patients currently for the prevention of CWP. They will receive usual care and there will be no restriction on what this can involve. CBT within the NHS is generally restricted to persons who have developed specific conditions rather than persons at risk of those conditions. We will however monitor care received, outwith the trial, in all participating subjects, and health care usage will be recorded in follow-up.

### Patient safety

There are unlikely to be major safety issues in terms of delivery of the CBT. However, if the therapists delivering CBT have any concerns, one of the investigators will be available to assess these. There will be a standard template for reporting concerns and recording of any action recommended.

### Withdrawal

Participants will have the option to withdraw from the treatment or the study at any time. Those withdrawing from the treatment will continue to be sent follow-up questionnaires unless they specifically request not to receive them. Failure of any participant to complete a follow-up questionnaire at any particular time-point will not be counted as a withdrawal unless the participant requests not to receive any further follow-ups.

### Statistical issues

#### Sample size

Our previous longitudinal study of onset of CWP (and subsequent replication) has suggested that 21 % of “high-risk” persons identified will develop CWP over the course of the next twelve months. Our previous data is based on persons with pain and at least 2 out of 3 other “risk factors”. There are no published studies of prevention of CWP on which to base our measure of effect. However in the MUSICIAN study some subjects, although reporting CWP at the screening survey, no longer had CWP at the enrolment interview. They were however still eligible to take part, provided they had regional pain. Therefore those subjects with regional pain provide a sub-population on which to base the likely effects of the tCBT. Amongst such subjects, those who received tCBT had a reduced odds of having CWP at the end of the study OR 0.5 95 % CI (0.2–1.4) compared to those in usual care.

Thus the study is powered on the ability of the current study to reduce the onset of CWP from 21 % to 12 %, with 90 % power and a 5 % significance level. We further assume, based on prior data, that 75 % of persons allocated to the tCBT arm will be adherent to the intervention, and that 80 % of all subjects will return the follow-up questionnaires to assess outcome.

Accordingly we require 473 subjects per arm that is a total of 946 subjects recruited. In MUSICIAN exactly 50 % of those found eligible and willing to consider taking part ultimately were randomised. A previous trial of a cognitive-behavioural intervention to prevent chronic pain found that 36 % of patients identified as eligible were recruited to the study [13]. If 80 % of eligible patients agreed to be contacted about taking part, this equates to 45 % of those eligible and willing to consider taking part being randomised - higher numbers for a clinical trial of CWP reflect the fact that this is a prevention trial rather than a treatment trial and may be less attractive to potential participants. Thus we aim to find a total of 2102 subjects who are eligible and willing to consider taking part. Assuming a participation rate to the survey of 30 %, that 1 in 4 people will be “at risk”, and (using data from MUSICIAN) that 80 % of people who return a questionnaire agree to consider taking part, we require to survey 35 037 persons.

### Statistical analysis

A pre-defined statistical analysis plan will be developed and signed off by the trial steering committee before undertaking any data analysis. Comparison between arms will be on an intention-to-treat basis (main analysis) with a per protocol sensitivity analysis.

Characteristics of the study participants in the two treatment arms will be described using simple summary statistics. Descriptive statistics will include mean and standard deviation for normally distributed continuous data, median and inter-quartile range for skewed continuous data and count and percentage for categorical data. No formal statistical comparisons will be made between baseline characteristics. Primary and secondary outcomes will be described at the three follow-up times: 3, 12 and 24 months, using appropriate summary statistics. The primary outcome is the between arm difference in the proportions of people developing CWP from baseline to follow-up. This comparison will be made using simple chi-squared tests at each follow-up time. Appropriate adjustment will be made for the stratification factor used in the randomisation (the number of non-pain “high-risk” factors that a participant reports at baseline) using multiple logistic regression.

Comparisons with appropriate hypothesis tests will be used for the secondary outcomes, pain, illness behaviour, somatic symptom reporting, sleep problems, quality of life and wellbeing, psychological distress, patient global impression of change measure and fatigue. Appropriate adjustment will be made for the stratification factor. Given the multiple secondary outcomes, the p-value used to denote statistical significance will reflect the multiple comparisons.

Mixed models analyses with an appropriate error structure will take into account the repeated assessment of the outcome data for the same patient across the three follow-up times. As part of sensitivity analyses, multiple imputation methods will be used, where appropriate, to address issues of missing data. However, these methods will not be applied if the use of imputation is contrary to specified rules for the relevant validated measurement scale.

### Study management and conduct

#### Trial steering committee

A trial steering committee will be established and comprise an independent chair who has expertise in both trials and CWP and two other independent members including a user representative who has had lived experience of CWP and a clinician working with people with CWP.

#### Inspection of records

Investigators and institutions involved in the study will permit study related monitoring and audits on behalf of the sponsor and REC. In the event of an audit or monitoring, the Investigator agrees to allow the representatives of the sponsor direct access to all study records and source documentation, and participant consent will be obtained for this.

#### Confidentiality

All evaluation forms, reports, and other records will be identified in a manner designed to maintain participant confidentiality. All records will be kept in a secure storage area with limited access.

#### Data protection

All Investigators and study site staff involved with this study will comply with the requirements of the Data Protection Act 1998 with regard to the collection, storage, processing and disclosure of personal information and will uphold the Act’s core principles. Computers used to collate the data will have limited access measures via user names and passwords. Published results will not contain any personal data that could allow identification of individual participants.

#### Study record retention

All study documentation will be kept for a minimum of 5 years from the protocol defined end of study point. When the minimum retention period has elapsed, study documentation will not be destroyed without permission from the sponsor.

#### End of study

The end of study is defined as data collection at 2 years from the last participant’s date of beginning treatment or dummy treatment start date.

#### Reporting, publication and notification of result

Ownership of the data arising from this study resides with the University of Aberdeen. On completion of the study, the study data will be analysed and tabulated, and a clinical study report will be prepared in accordance with ICH guidelines. The clinical study report will be used for publication and presentation at scientific meetings. Investigators have the right to publish orally or in writing the results of the study.

#### Insurance and indemnity

The University of Aberdeen is sponsoring the study. The University of Aberdeen will obtain and hold a policy of Public Liability Insurance of legal liabilities arising from the study. Where the study involves University of Aberdeen staff undertaking clinical research of NHS patients, such staff will hold honorary contracts with Grampian Health Board. The sponsor does not provide study participants with indemnity in relation to participation in the study but has insurance for legal liability as described above.

## Discussion

The study will be first aimed at preventing the development of fibromyalgia or chronic widespread pain. The results of the study will help to inform future treatments for the prevention of chronic pain, and aetiological models of its development.

### Ethics approval and consent to participate

The NHS Research Ethics Committee (REC)-Central Booking Service (CBS) was used to book the study for full REC review. The CBS offers the first available meeting in the UK, and so REC South West–Cornwall & Plymouth gave approval to the study rather than a Scottish committee. The study was approved by the REC South West–Cornwall & Plymouth on 8 February 2016, and the REC Reference is 16/SW/0019. As stipulated by the ethics committee management approval from all NHS sites has been obtained.

All participants in the trial will first read an information sheet and return by post a signed consent form, before being contacted by a researcher by telephone and given the opportunity to ask questions about the study and confirm their consent to take part. Only after having both returned a signed consent form and confirmed consent over the phone will a person be considered as having given consent and recruited to the trial.

### Consent for publication

Not applicable.

### Availability of data and material

There are no plans to place data in a repository at the current time.

## Abbreviations

CBS: Central Booking Service
CBT: Cognitive Behavioural Therapy
CWP: Chronic Widespread Pain
GP: General Practitioner
NHS: National Health Service
REC: Research Ethics Committee
SPCRN: Scottish Primary Care Research Network
tCBT: Telephone-delivered Cognitive Behavioural Therapy
UK: United Kingdom
